# A Dual-Band Guided Laser Absorber Based on Plasmonic Resonance and Fabry-Pérot Resonance

**DOI:** 10.3390/nano12162751

**Published:** 2022-08-11

**Authors:** Xinye Liao, Junxiang Zeng, Yunxiang Zhang, Xin He, Junbo Yang

**Affiliations:** 1 Undergraduate School, National University of Defense Technology, Changsha 410073, China; 2 Department of Physics, National University of Defense Technology, Changsha 410073, China

**Keywords:** metamaterial, plasmonic resonance, Fabry–Pérot resonance, dual-band, guided lasers

## Abstract

We numerically investigated a dual-band metamaterial absorber based on the combination of plasmonic resonance and Fabry–Pérot (FP) resonance, which can achieve near-unity absorption for guided lasers. The absorber is constructed by a three-layer metal-insulator-metal (MIM) periodic configuration. In each unit cell, there is a gold-silicon cross on a thin silicon layer and a bottom nickel film. Numerical results show that, at normal incidence, the structure strongly absorbs light at wavelengths of 1.064 μm and 10.6 μm, with absorption rates higher than 94%. It is revealed that the two absorption peaks result from FP resonance in the thin silicon layer and plasmonic resonance in the cross, respectively. In addition, the absorber is polarization insensitive and is tolerant to the incident angle. The proposed combination of different resonances has the advantage of easily producing double absorption peaks with very large wavelength differences, and provides a new approach to the design of metamaterial absorbers.

## 1. Introduction

Absorbing materials are a class of functional materials that can effectively absorb electromagnetic waves and significantly reduce reflection. Traditional absorbing materials include ferrite [1], ultrafine metallic powder [2], polycrystalline iron fiber [3], plasma absorbing material [4], and so on. They can operate at various frequencies, such as microwaves, millimeter waves, terahertz, and infrared, and have been widely used in many fields.

In the visible and infrared wavelengths, traditional absorbing materials are generally thick and heavy, and have poor stability. It is an issue, especially in military applications where thin and light-absorbing materials are very necessary. For this reason, absorbing materials composed of a few layers and micro- and nano-structures have recently attracted great attention. Using micro- and nano-structures with characteristic lengths smaller than or close to the wavelength of incident light [5], it is also possible to achieve special physical properties that traditional materials do not have, such as negative refractive index [6,7], negative magnetic permeability [8], negative dielectric constant [9], etc. Thus, micro- and nano-structure-based materials (including metamaterials) are potentially useful in stealth camouflage [9,10], polarization detection [11], perfect absorber [12], super-lenses [13], nano-heater [14], anti-icing effects [15], and so on.

In the past few years, micro- and nano-structure-based materials have developed rapidly. These types of absorbers operating at visible and infrared wavelengths have been demonstrated. Moreover, their absorption functions have gradually expanded from single-band [16] to multi-band [17,18]. Take the metamaterial absorber as an example; the main principle behind its effective absorption at a certain wavelength is plasmonic resonance (typically excited by incident waves in an MIM ‘sandwich’ structure [19]). The shape, size, and period of micro- and nano-structures can significantly influence the plasmonic resonance [20]. Based on this feature, resonant characteristics can be designed by adjusting the geometry, size, and distribution of the structural units.

Focusing our attention on military ‘stealth’ applications, the absorption of laser light at 1.064 μm and 10.6 μm wavelengths is highly desirable because these two bands are widely used in laser-guided equipment. However, to absorb laser light at the two wavelengths simultaneously and efficiently, there are still problems to be solved. The main way for metamaterial absorbers to realize multi-band absorption is to arrange or combine structural units according to certain rules, such as planar combination [21], vertical stacking [22], and structural splicing [23]. These methods may not be effective because the two wavelengths are extremely different. Conventional design tends to cause the resonance at the shorter wavelength to behave as propagating plasmons. As a result, the absorber will be sensitive to the angle of incidence. Some researchers have tried to use the Helmholtz cavity [24], but the structure is relatively difficult to fabricate. On the contrary, FP resonance is simpler in structure and easier to manufacture. Moreover, if a high refractive index dielectric is used as the intermediate layer, it is convenient to strongly confine the resonant electromagnetic field and form an absorption characteristic that is insensitive to the angle of incidence.

In this work, we attempted to combine an FP cavity with a plasmonic resonator to achieve efficient absorption of laser light at the 1.064 μm and 10.6 μm wavelengths. FP cavities have the advantage of being simple and easy to fabricate. Plasmonic resonators have the advantage of being insensitive to the incident angle. To explain the absorption mechanism of our structure, the electromagnetic field distribution and energy loss were numerically investigated. The dependence on incident polarization and angle were simulated. In addition, considering the fabrication error, the influence of geometric parameters on the absorption performance was discussed.

## 2. Structure Design and Performance

**Figure 1:**
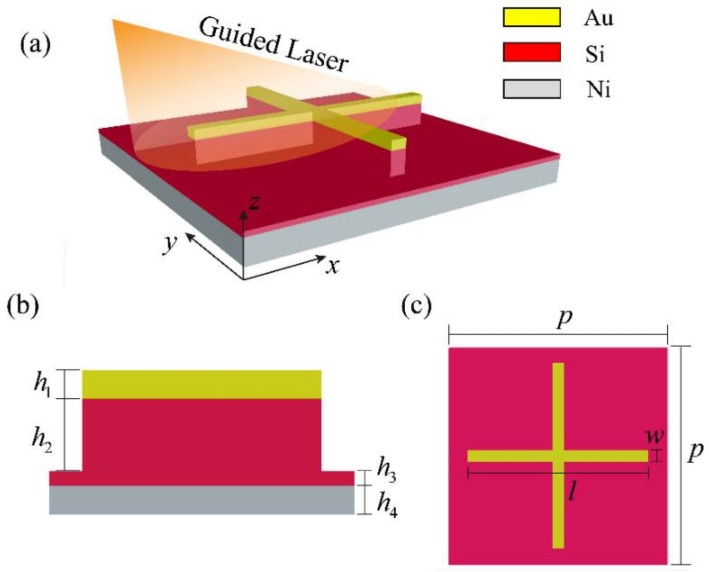
Schematic diagram of the proposed dual-band guided laser absorber, where the 3D view (**a**), side view (**b**), and top view (**c**) of each unit cell are shown.

Figure 1 illustrates the structure of the proposed metamaterial absorber. It is an MIM periodic configuration with three functional layers. In each unit cell of the absorber, there is a gold-silicon cross on a thin silicon layer and a bottom nickel film, as shown in Figure 1a. The gold cross and the silicon cross have thicknesses of $h_{1}$ and $h_{2}$, respectively. Under the gold-silicon cross, the thin silicon layer has a thickness of $h_{3}$, and the bottom nickel film has a thickness of $h_{4}$, as shown in Figure 1b. In Figure 1c, it is seen that the absorber has a period of p, and the gold-silicon cross in each unit cell has a length of l and a width of w. In order to achieve near-unity absorbance, finite-difference time-domain (FDTD) simulations were performed to optimize the geometric parameters. In the simulations, optical parameters of gold, silicon, and nickel in [25] were employed.

The gold-silicon-nickel cavity is designed to function as a leaky resonator [26,27]. According to the critical coupling theory and the single-resonance model, the following equation can be obtained.

(1) $$Z(\omega )=\frac{\omega_{ie}}{-i(\omega -\omega_{0})+\omega_{i0}}$$

where ω=2πc/λ is the frequency of the incidence, Z is the impedance of the resonator, $\omega_{ie}$ is the radiative damping/coupling rate, and $\omega_{i0}$ is the resistive damping rate. When the incident wave excites the eigenmodes of the resonator through radiative coupling, the absorption rate A(ω) can be determined by

(2) $$A(\omega )=1-{\left|\frac{Z(\omega )-1}{Z(\omega )+1}\right|}^{2}$$

The thin silicon layer is designed to function as an FP resonant layer. Taking into account the half-wave loss, the destructive interference of reflection occurs when

(3) $$2n_{eff}h_{3}=k\lambda ,k=1,2,3,\ldots \ldots$$

where λ is the resonance wavelength and $n_{eff}$ is the effective refractive as a function of λ. Note that there is k=1 in our structure to make the FP resonant layer as thin as possible.

**Figure 2:**
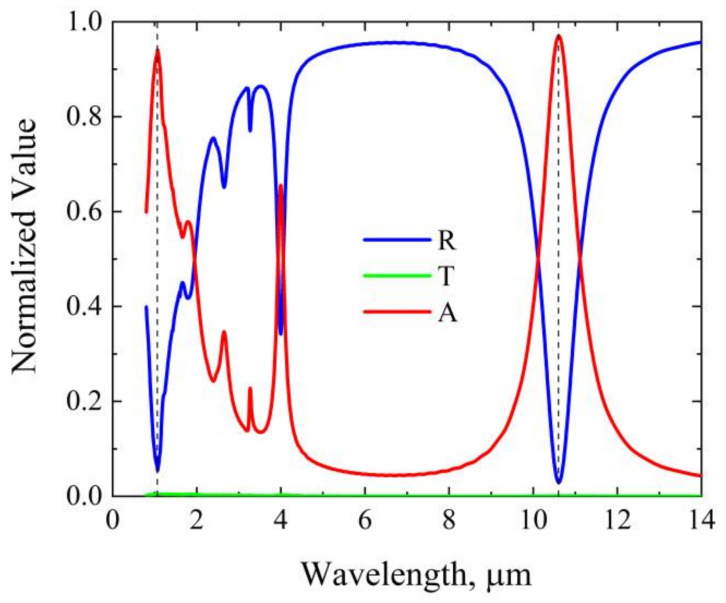
Optimized absorbance (A), reflectance (R), and transmittance (T) at normal incidence. The vertical dashed lines mark the wavelengths of 1.064 μm and 10.6 μm, respectively.

Figure 2 shows the calculated absorbance, reflectance, and transmittance of the absorber at normal incidence. The geometric parameters are as follows: p = 3.2 μm, l = 2.51 μm, w = 0.15 μm, $h_{1}$ = 0.1 μm, $h_{2}$ = 0.25 μm, $h_{3}$ = 0.05 μm, and $h_{4}$ = 0.1 μm. It can be seen that, in the investigated spectral region, the transmittance is nearly zero. The reflectance has several spectral dips. In particular, there are two reflection dips centered at wavelengths of 1.064 μm and 10.6 μm, respectively. The absorbance of the absorber satisfies A=1−R−T, where R and T are the reflectance and transmittance, respectively. Thus, we can see that the absorbance at wavelengths of 1.064 μm and 10.6 μm reach ~94% and ~97%, respectively. These indicate that the proposed absorber can achieve efficient absorption of the most widely used guided lasers.

## 3. Mechanism and Discussion

**Figure 3:**
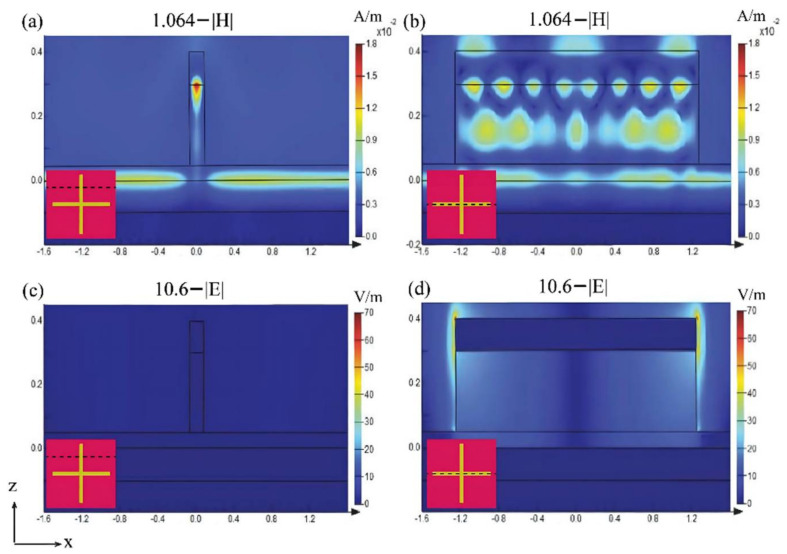
Cross-sectional view of electromagnetic field distributions at wavelengths of 1.064 μm and 10.6 μm. (**a**,**b**) Show the magnetic field distributions at the wavelength of 1.064 μm, corresponding to the cross-sections in the insets, respectively. (**c**,**d**) Show the electric field distributions at the wavelength of 10.6 μm. Note that the cross-sections in (**a**,**c**) are the same, and the cross-sections in (**b**,**d**) are the same.

First, to explain the absorption mechanism of our dual-band guided laser absorber, we investigated electromagnetic field distributions at wavelengths of 1.064 μm and 10.6 μm. The results in each unit cell are plotted in Figure 3. For the 1.064 μm wavelength, the magnetic field is mainly confined within the thin silicon layer under the gold-silicon cross, as shown in Figure 3a. This implies a typical FP resonance, where the silicon surface and the silicon-nickel interface act as mirrors. In Figure 3a,b, we see that there is also a portion of the magnetic field confined within (bonded to) the gold-silicon cross. However, due to the narrowness of the cross, this portion of the magnetic field has little effect on the absorption of the 1.064 μm wavelength light. For the 10.6 μm wavelength, the electric field is almost totally confined between the gold cross and nickel film, and is especially strong at the ends of the gold cross. This implies a typical plasmonic resonance, as shown in Figure 3c,d. The results suggest that the absorption at the wavelength of 1.064 μm is mainly attributed to the FP resonance, whereas the absorption at the wavelength of 10.6 μm is attributed to the plasmonic resonance.

**Figure 4:**
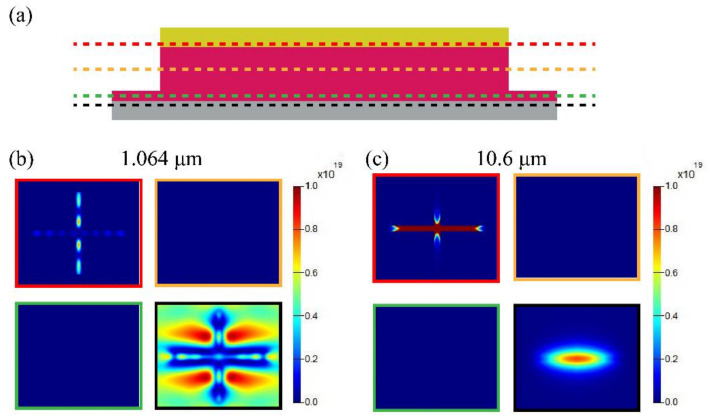
Energy dissipation distributions in the absorber at wavelengths of 1.064 μm and 10.6 μm. (**a**) Shows the locations of different cross-sections represented by dashed lines in different colors. (**b**) Shows energy dissipation at the wavelength of 1.064 μm. (**c**) Shows energy dissipation at the wavelength of 10.6 μm. These results were obtained under the assumption that the polarization of incidence is along the *x*-axis. The different colored borders in (**b**,**c**) correspond to the cross-sections shown in (**a**).

Further understanding can be gleaned from Figure 4, where the energy loss at the 1.064 μm and 10.6 μm wavelengths is illustrated. We have chosen four planes in the absorber to calculate energy loss, as shown in Figure 4a. For the 1.064 μm wavelength, Figure 4b indicates that the energy is mainly dissipated in the bottom nickel film as an ohmic loss. Meanwhile, the energy loss in the gold cross is significantly smaller than that in the bottom nickel film. On the contrary, there is almost no loss in the silicon cross and the silicon layer. These are consistent with the above-mentioned FP resonance. For the 10.6 μm wavelength, the energy dissipation also occurs in the two metallic layers as an ohmic loss, but the energy loss in the gold cross is very large, as shown in Figure 4c. Considering the field distribution in Figure 3d, this is clearly consistent with the characteristics of plasmonic resonance.

**Figure 5:**
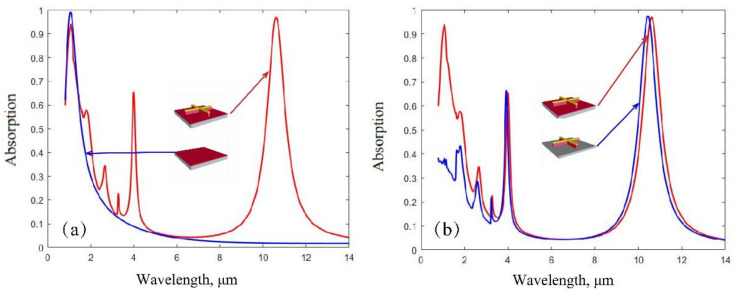
Absorption spectra when the structure contains only the FP resonator (**a**) or the plasmonic resonator (**b**). Insets show the corresponding structure in each unit cell. The geometric parameters are kept unchanged. For comparison, the absorption spectrum in Figure 2 is also plotted.

In order to verify the above explanation, we performed simulations assuming that the structure on the bottom nickel film contains only the thin silicon layer or the gold-silicon cross. The obtained absorption spectra are shown in Figure 5. In Figure 5a, it is obvious that the absorption peak at the wavelength of 1.064 μm results from the thin silicon layer, that is, the FP resonance. However, Figure 5b indicates that the absorption peak at the wavelength of 10.6 μm results from the gold-silicon cross, that is, the plasmonic resonance. Thus, our dual-band guided laser absorber is enabled by the combination of a plasmonic resonator and an FP cavity.

**Figure 6:**
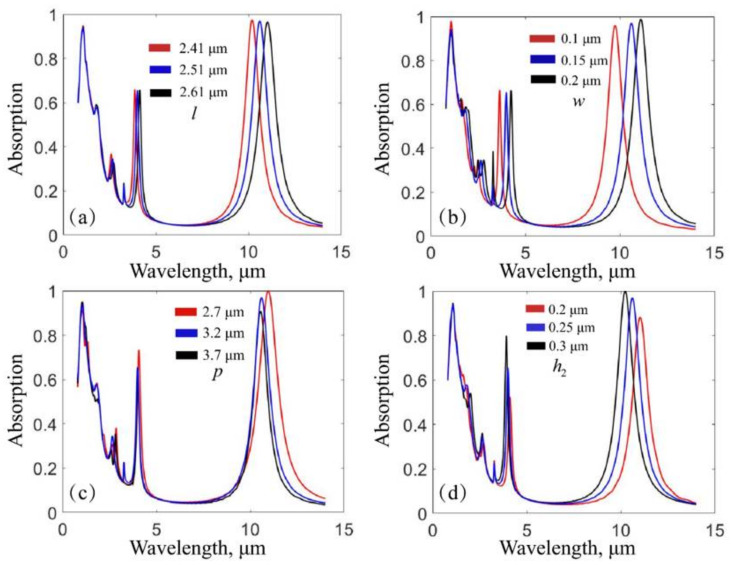
Absorption spectra when independently changing the length of the gold-silicon cross l (**a**), the width of the gold-silicon cross w (**b**), the structural period p (**c**), and the thickness of the silicon cross $h_{2}$ (**d**).

Then, the resonance wavelength of the upper plasmonic resonator is not only related to the shape of the resonator, but also depends on the thickness of the dielectric and the period of the structure. To take this into account, we studied the variation of the two absorption peaks by changing the values of geometric parameters, as shown in Figure 6. In Figure 6a, when the length of the gold-silicon cross increases, the absorption peak at the 1.064 μm wavelength is almost unchanged, and the absorption peak at the 10.6 μm wavelength exhibits a significant redshift. In Figure 6b–d, similar phenomena can be observed when the width of the gold-silicon cross increases, the period of the absorber decreases, and the thickness of the silicon cross decreases.

The observed invariance of the 1.064 μm wavelength peak is due to the peak resulting from FP resonance. The observed redshift of the 10.6 μm wavelength peak is due to the fact that the resistive damping rate in Equation (1) decreases as the size of the gold-silicon cross increases, thus, increasing the absorption peak wavelength [25]. At the same time, we see that the absorbance at the 10.6 μm wavelength is affected by changes in the geometric parameters. The reason is that geometric changes can vary the radiative damping/coupling rate in Equation (1), and thus the absorption rate in Equation (2). These results imply that we are able to tune the longer wavelength peak without affecting the peak at the 1.064 μm wavelength.

**Figure 7:**
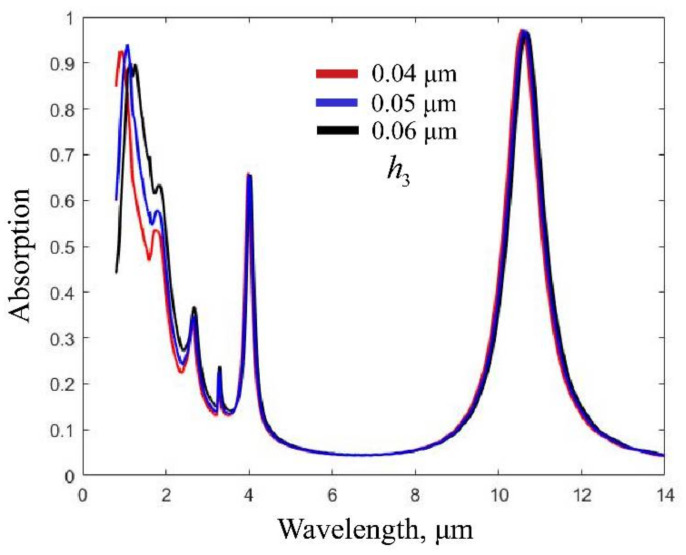
Absorption spectra with different thicknesses of the thin silicon layer.

Figure 7 shows the spectral response of the absorber when changing the thickness of the thin silicon layer (i.e., $h_{3}$). It can be clearly seen that the absorption peak at the 10.6 μm wavelength is almost invariant, whereas the absorption peak at the 1.064 μm wavelength changes greatly. It is easy to understand because the main role of the thin silicon layer is to form an FP cavity, which absorbs light at the 1.064 μm wavelength. We can also observe an obvious redshift of the shorter wavelength peak with increasing thickness of the thin silicon layer, as described in Equation (3). By adjusting the thickness of the thin silicon layer, we were able to tune the shorter wavelength peak without affecting the peak at the 10.6 μm wavelength.

**Figure 8:**
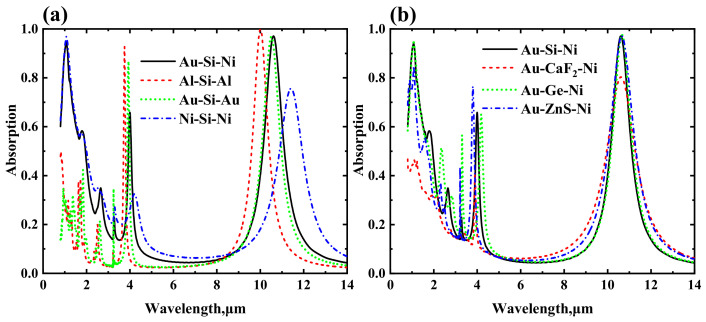
Absorption spectra when choosing different materials. (**a**) Changing the top and bottom metals. (**b**) Changing the intermediate dielectric.

Figure 8 illustrates the calculated absorption spectra when choosing different materials. In Figure 8a, the top and bottom metals are changed, while the dielectric remains silicon. For the Al-Si-Al and Au-Si-Au structures, there is a peak at approximately 10.6 μm wavelength, whereas the peak at the 1.064 μm wavelength almost disappears. For the Ni-Si-Ni structure, the peak at the 1.064 μm wavelength exists, whereas the absorption rate at approximately 10.6 μm decreases significantly. Compared with the absorption spectra of the Au-Si-Ni structure, it is indicated that the bottom metal plays a crucial role in producing the peak at the 1.064 μm wavelength, and it is advantageous to use metals with relatively high losses. Using different metals affects the peak at approximately 10.6 μm wavelength to some extent, but not significantly. The observed red or blue shift can be compensated for by adjusting the geometric parameters of the cross.

In Figure 8b, the dielectric is varied, while the top and bottom metals are kept unchanged. For ease of comparison, the geometric parameters have been re-optimized. For the Au-Si-Ni and Au-Ge-Ni structures, both of the peaks can have high absorbance. It is clearly seen that if the refractive index of the dielectric is relatively small, the absorbance at 1.064 μm wavelength is much lower. A similar phenomenon is shown at the 1.064 μm wavelength. This suggests that a high-index dielectric, such as Si or Ge, is needed in our dual-band laser absorber.

**Figure 9:**
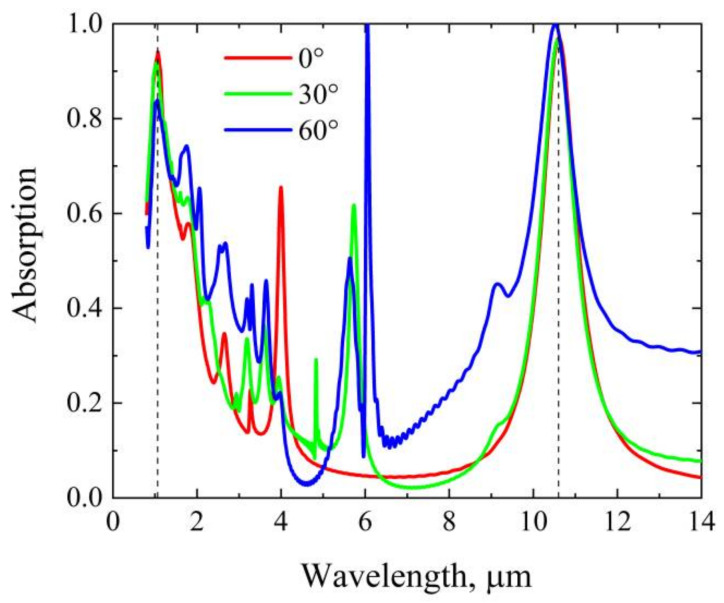
Absorption spectra at different angles of incidence. The vertical dashed lines mark the wavelengths of 1.064 μm and 10.6 μm, respectively.

Finally, in practical applications, the spectral response of the absorber to the polarization and angle of incidence plays an important role. Owing to the nature of FP resonance and the geometric symmetry of the gold-silicon cross in each unit cell, the designed absorber is certainly polarization insensitive. Figure 9 shows simulated absorption spectra at different angles of incidence. As can be seen, at oblique incidence, the absorbance at the 1.064 μm wavelength is reduced, while the absorption peak at the 10.6 μm wavelength only shifts a little. However, at incident angles of up to 60 degrees, the absorbance of the two absorption bands is still higher than 0.84. The results show that the absorber can achieve wide-angle absorption of guided lasers.

## 4. Conclusions

We have numerically presented a dual-band metamaterial absorber based on the combination of plasmonic resonance and FP resonance. The absorber consists of periodic gold-on-silicon crosses seated on a thin silicon layer and a bottom nickel film. Based on FDTD simulations, we show that near-unity absorption can be achieved at wavelengths of 1.064 μm and 10.6 μm. At normal incidence, the absorbance at the two wavelengths is as high as ~94% and ~97%. It is found that the two absorption peaks result from FP resonance and plasmonic resonance, respectively. Independent tuning of the peaks is possible by adjusting the thickness of the thin silicon layer and the geometric parameters of the gold-silicon cross. Moreover, the absorber is polarization insensitive and maintains >80% absorbance even at incidence angles of up to 60 degrees. The advantage of this design is that it is easy to form double absorption peaks with a large wavelength difference. It can also be extended to other frequencies for perfect absorption, so it has potential applications in guided laser stealth, optical detection, military camouflage, and so on.

## Data Availability

The data presented in this study are available on request from the corresponding author.
